# Methyl 3-Bromo-4,5-dihydroxybenzoate Attenuates Inflammatory Bowel Disease by Regulating TLR/NF-κB Pathways

**DOI:** 10.3390/md23010047

**Published:** 2025-01-19

**Authors:** Jing Huang, Lei Li, Liyan Xu, Lixin Feng, Yuxin Wang, Attila Gabor SIK, Meng Jin, Rongchun Wang, Kechun Liu, Xiaobin Li

**Affiliations:** 1 Biology Institute, Qilu University of Technology (Shandong Academy of Sciences), Jinan 250103, China; huangjing0126@126.com (J.H.); ll1061602375@163.com (L.L.); 15966535461@163.com (L.X.); flixin2022@163.com (L.F.); 17860282390@163.com (Y.W.); sik.attila@pte.hu (A.G.S.); mjin1985@hotmail.com (M.J.); 2 Engineering Research Center of Zebrafish Models for Human Diseases and Drug Screening of Shandong Province, Jinan 250103, China; 3 Institute of Physiology, Medical School, University of Pecs, H-7624 Pecs, Hungary; 4 University Research and Innovation Center, Obuda University, H-1034 Budapest, Hungary

**Keywords:** methyl 3-bromo-4,5-dihydroxybenzoate, zebrafish, TNBS, inflammatory bowel disease, NF-κB pathway

## Abstract

Inflammatory bowel disease (IBD) is characterized by uncontrolled, chronic relapsing inflammation in the gastrointestinal tract and has become a global healthcare problem. Here, we aimed to illustrate the anti-inflammatory activity and the underlying mechanism of methyl 3-bromo-4,5-dihydroxybenzoate (MBD), a compound derived from marine organisms, especially in IBD, using a zebrafish model. The results indicated that MBD could inhibit the inflammatory responses induced by CuSO_4_, tail amputation and LPS in zebrafish. Furthermore, MBD notably inhibited the intestinal migration of immune cells, enhanced the integrity of the gut mucosal barrier and improved intestinal peristalsis function in a zebrafish IBD model induced by trinitro-benzene-sulfonic acid (TNBS). In addition, MBD could inhibit ROS elevation induced by TNBS. Network pharmacology analysis, molecular docking, transcriptomics sequencing and RT-PCR were conducted to investigate the potential mechanism. The results showed that MBD could regulate the TLR/NF-κB pathways by inhibiting the mRNA expression of *TNF-α*, *NF-κB*, *IL-1*, *IL-1β*, *IL6*, *AP1*, *IFNγ*, *IKKβ*, *MyD88*, *STAT3*, *TRAF1*, *TRAF6*, *NLRP3*, *NOD2*, *TLR3* and *TLR4*, and promoting the mRNA expression of *IL4*, *IκBα* and *Bcl-2.* In conclusion, these findings indicate that MBD could be a potential candidate for the treatment of IBD.

## 1. Introduction

Inflammation is a complicated process and essential for the body against tissue/cell damage or infection [1]. However, uncontrolled and chronic inflammations are harmful to the body, and are closely related to various diseases, such as cancers, cardiovascular diseases and inflammatory bowel disease (IBD) [2,3,4]. IBD has become a worldwide health problem, and is characterized by uncontrolled and excessive inflammation in the intestine. IBD mainly includes Crohn’s disease (CD) and ulcerative colitis (UC) [5,6]. When IBD occurs, the gut barrier is damaged with a reduction in goblet cells and junction proteins, which can lead to the infiltration of harmful bacteria into the intestine, and then cause inflammation. It can further lead to weight loss, pain and even rectal bleeding. Oxidative damage and even apoptosis are closely involved in the process of IBD [7,8]. Excessive ROS can cause damage to DNA, protein and membrane lipids and then cause apoptosis of the cells, which is essential to the gut barrier. A lot of drugs have been developed and applied to treat IBD [7,8]. However, unsatisfactory therapeutic effects and easy recurrence remain problems for IBD treatment. Therefore, more attention should be paid to searching for green and efficient drugs against IBD.

Rich marine organisms, which live in different environments, such as those with high pressure, high salinity, low oxygen content and low temperature, provide various natural chemicals that are different from those of terrestrial organisms [9]. Recent years, abundant novel chemicals, such as steroids, alkaloids, polyketides and peptides, have been identified from marine organisms, and have various bioactivities, including antitumor, antiviral, antibacterial and anti-inflammatory activities [10]. Bromophenols, common secondary metabolites, can be isolated from marine organisms, including marine algae, sponges, ascidians and so on. Bromophenols exhibit various biological activities, such as anticancer, antidiabetic, antimicrobial and anti-inflammatory [11]. Methyl 3-bromo-4,5-dihydroxybenzoate (MBD), one of the bromophenols, is derived from Red Alga *Rhodomela confervoides* [12]. However, its biological activity has not been fully investigated. Here, its anti-inflammatory activity, especially in IBD was studied in a zebrafish model.

Recent years, zebrafish (*Danio rerio*), a small tropical freshwater fish, has been widely used as a powerful animal model in the immunological field [13,14]. It has various advantages, such as small size, high productive rate, in vitro fertilization and rapid growth [13,14]. In addition, at the early developing stage, the embryo/larva is transparent, making it easy to perform real-time observations of the organ or specific cells, such as immune cells, nerve cells labeled with florescent proteins in the transgenic zebrafish line [15,16]. The dynamic inflammation process also can be easily observed under a fluorescence microscope. Inflammatory zebrafish models, which are induced by CuSO_4_, lipopolysaccharide (LPS), tail-cutting, bacterial infection or TNBS, have been widely used to study inflammatory mechanisms or screen compounds with anti-inflammatory activity [16,17,18]. Thus, a zebrafish model was used to investigate the anti-inflammatory activity and the underlying mechanism of MBD against IBD.

Here, anti-inflammatory activity was evaluated using zebrafish models induced by CuSO_4_, LPS and tail-cutting. Furthermore, the relieving effect of MBD against IBD was studied in a TNBS-induced zebrafish model and the underlying mechanism was explored using network pharmacology, mRNA sequencing and an RT-PCR test.

## 2. Results

### 2.1. The Activity of MBD Against Inflammation Induced by CuSO_4_, LPS or Tail-Cutting

**Figure 1:**
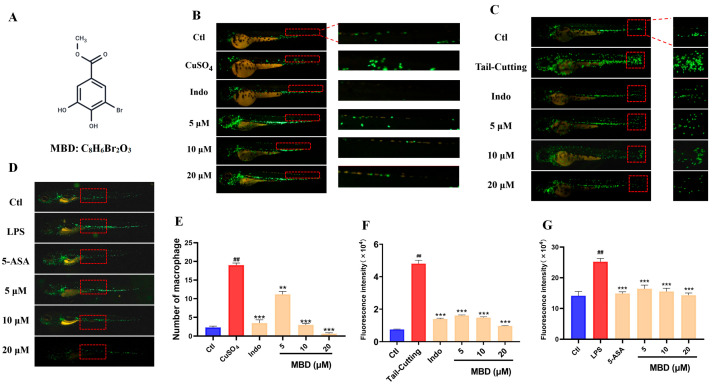
The anti-inflammatory effect of MBD against CuSO_4_, LPS or tail-cutting. (**A**) The chemical structure of MBD. Representative images of the anti-inflammatory effects in the CuSO_4_-induced acute inflammation (**B**), tail-cutting-induced mechanic damage inflammation (**C**) and LPS-induced systemic inflammation models (**D**). (**E**–**G**) show the statistical analysis of (**B**–**D**). The migrated and aggregated immune cells shown in the red area were used for the analysis. ## *p* ≤ 0.01 vs. Ctl; ** *p* ≤ 0.01 and *** *p* ≤ 0.001 vs. model.

CuSO_4_ can induce acute inflammation by causing damage to facial nerve cells, leading to the migration of the immune cells [19]. Compared with the control (Ctl) group, CuSO_4_ caused a significant increase in the immune cells that migrated to the damaged area, while MBD could inhibit the migration of immune cells in the *Tg(zlyz:EGFP)* zebrafish line (Figure 1B).

Tail-cutting can induce inflammation by damaging the zebrafish tail [20]. As shown in Figure 1C, much more migrated immune cells were shown in the damaged area, while fewer immune cells were located in the damaged area in the MBD treated groups.

LPS, derived from bacteria, can cause systemic zebrafish inflammation, leading to a remarkable increase in immune cells (Figure 1D) [21]. After treatment with MBD, the number elevation of immune cells could be inhibited. These results indicated that MBD had an apparent anti-inflammatory effect.

### 2.2. MBD Relieved TNBS-Induced IBD

**Figure 2:**
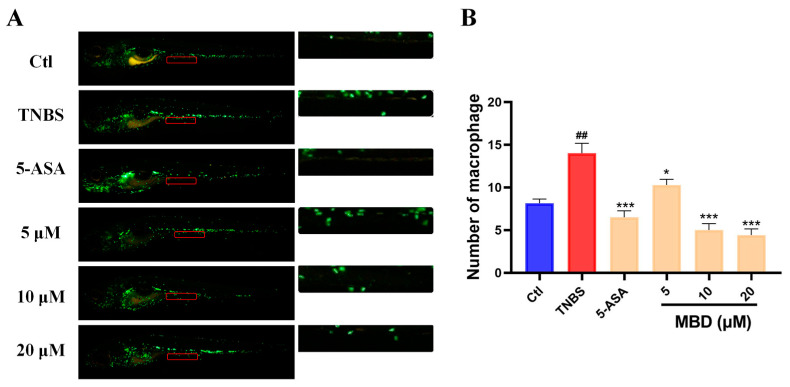
The anti-inflammatory effect of MBD in the zebrafish IBD model induced by TNBS. (**A**) A representative image. (**B**) The statistical analysis was conducted on the migrated immune cells shown in indicated red area. ## *p* ≤ 0.01 vs. Ctl; * *p* ≤ 0.05, *** *p* ≤ 0.001 vs. model.

TNBS is widely used to establish IBD in zebrafish or mice by disrupting the gut mucosal barrier, activating inflammatory-related pathways and then increasing the intestinal migration of immune cells [22,23]. TNBS increased the migration of immune cells to the intestine compared with the control groups, while MBD could inhibit the intestinal location of immune cells compared with IBD group (Figure 2).

### 2.3. MBD Enhanced the Peristaltic Ability of the Intestine

**Figure 3:**
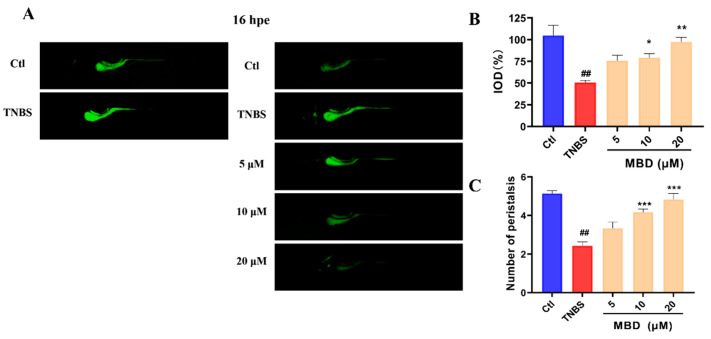
The protective effect of MBD on intestinal peristalsis and efflux activity. The zebrafish was stained with calcein solution for 16 h. (**A**) Images representative of the zebrafish stained with calcein solution at 0 h and 16 h. (**B**) The statistical analysis of the integrated optical density (IOD) parameter of the calcein staining. (**C**) The peristalsis frequency 1 min after calcein staining. ## *p* ≤ 0.01 vs. Ctl; * *p* ≤ 0.05 vs. model, ** *p* ≤ 0.01 vs. model, *** *p* ≤ 0.001 vs. model.

Since the epithelial cells of the intestine have a high rate of endocytosis, the peristaltic ability of the zebrafish intestine can be detected by live staining using calcein dye. The exocytosis of the calcein dye indicated the peristaltic ability and the intestine. As shown in Figure 3, after 16 hpe (hours post exposure), the fluorescence intensity of the calcein dye in the TNBS group was higher than that in the control group, while it was lower in the MBD group than in the TNBS group. Furthermore, the peristalsis number in 1 min in the MBD group was higher than that in TNBS group. These results indicated that intestine function could be improved by MBD.

### 2.4. MBD Enhanced the Integrity of the Intestinal Structure

**Figure 4:**
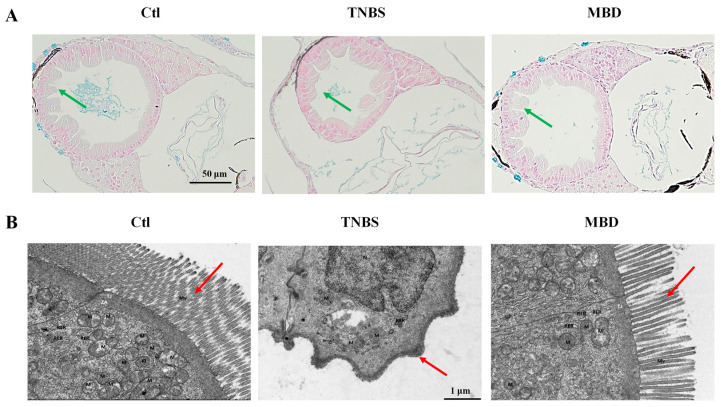
MBD enhanced the integrity of the intestinal structure. Images representative of HE staining (**A**) and TEM (**B**). Green arrows indicate the integrity of intestinal mucosal epithelial cells, and red arrows indicate microvilli. M, mitochondria; RER, rough endoplasmic reticulum; Mv, microvilli.

The integrity of the intestinal structure was detected by hematoxylin–eosin (HE) staining and by transmission electron microscopy (TEM) after MBD exposure. The HE staining results indicated that there was less integrity of the intestinal structure in TNBS group compared to the control group, whereas there was more integrity of the intestinal structure in the MBD group compared to the TNBS group (Figure 4A). Furthermore, from the TEM picture, it is apparent that there were more microvilli and there was less disruption of mitochondria in the MBD group than in the TNBS group (Figure 4B). These results indicated that MBD could inhibit inflammation by protecting the gut barrier.

### 2.5. MBD Reduced ROS Elevation Induced by TNBS

**Figure 5:**
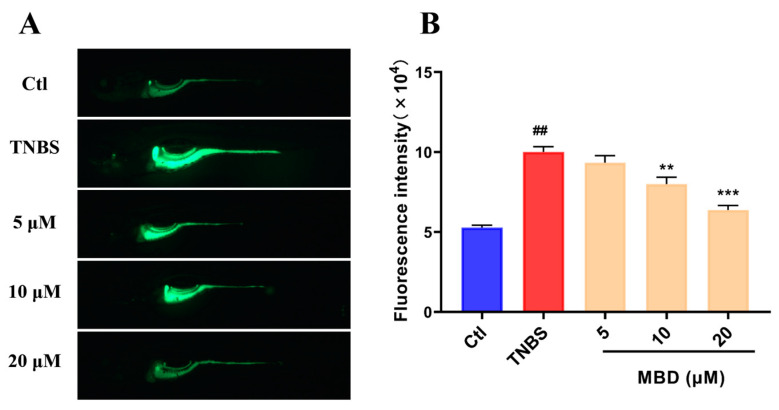
The inhibition of ROS elevation induced by TNBS. (**A**) Representative florescence images of ROS staining. (**B**) The statistical analysis of fluorescence IOD. ## *p* ≤ 0.01 vs. Ctl; ** *p* ≤ 0.01, *** *p* ≤ 0.001 vs. model.

To further investigate the anti-inflammatory mechanism of MBD, the ROS level, which was closely related to the occurrence and development of inflammation, was tested after MBD exposure. As shown in Figure 5, the ROS level significantly increased after TNBS treatment, whereas ROS elevation was remarkably inhibited by MBD treatment (Figure 5).

### 2.6. Network Pharmacology Analysis

**Figure 6:**
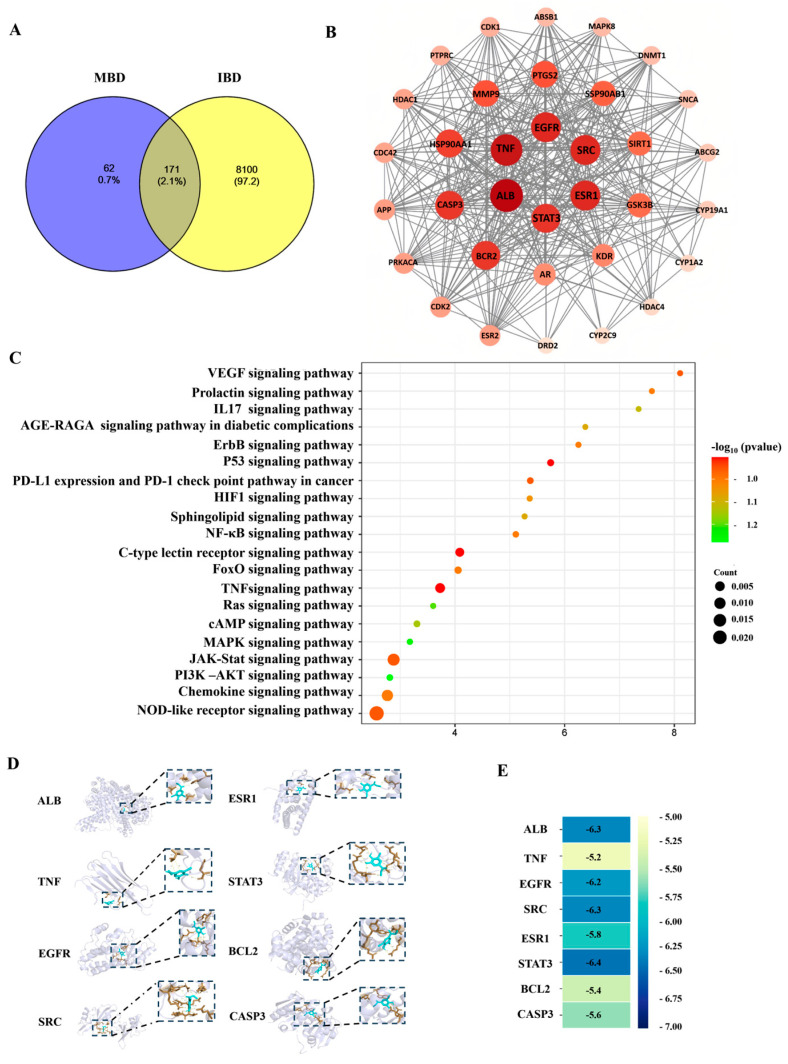
Network pharmacology analysis. (**A**) The potential targets of MBD (233 genes) and IBD (8271 genes). There are 171 gene targets overlapped with IBD and MBD potential targets. (**B**) The PPI network of interaction proteins. (**C**) The KEGG pathways. (**D**) Molecular docking images of MBD with the potential targets. (**E**) The combining thermal energy between MBD and the targets.

The network pharmacology analysis results showed that 233 potential targets which might interact with MBD, and 8271 potential targets related to IBD, were found in the PharmMapper (Version 2017), SEA and STITCH databases (Figure 6A). There were 171 genes shared by MBD and IBD. Figure 6B shows the key targets analyzed and mapped by STRING and Cytoscape 3.6.1 software. ALB, TNF, EGFR, SAC, ESR1, STAT3, BCL2 and CASP3 were listed as the first eight targets. The KEGG results showed that the main pathways related to MBD and IBD were primarily correlated with the NOD pathway, JAK-STAT pathway, TNF pathway, P53 pathway and chemokine-related pathway (Figure 6C). Molecular docking was used to test MBD and these eight targets. The molecular docking results showed that MBD could interact with these proteins, and the docking site and combining thermal energy are shown in Figure 6D,E.

### 2.7. Transcriptome Analysis

**Figure 7:**
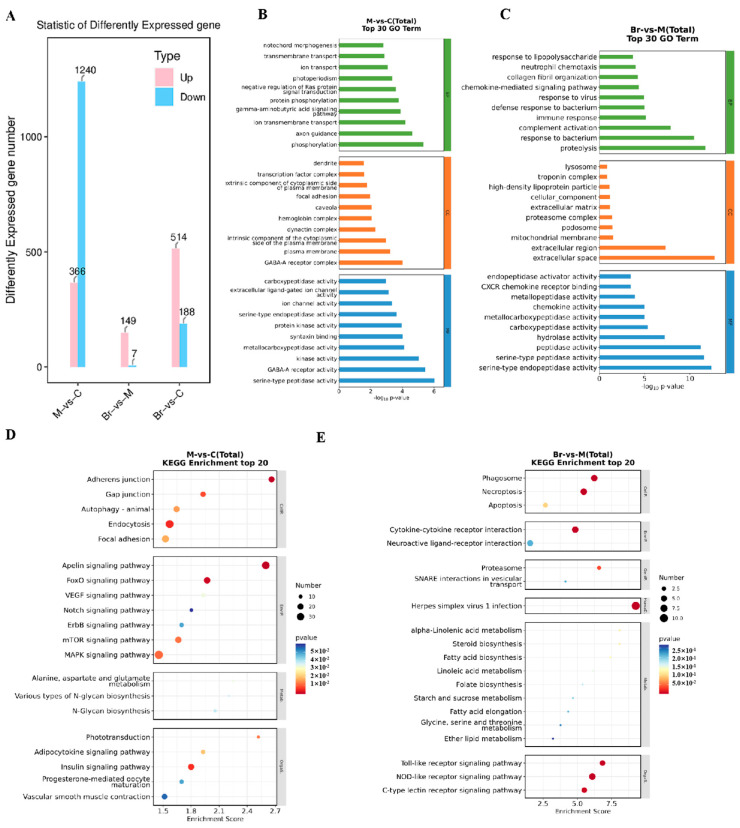
Transcriptome analysis. (**A**) DEGs between the TNBS, control and/or MBD groups, respectively. The GO analysis between TNBS vs. control group (**B**) and MBD vs. TNBS group (**C**). The biological processes, cellular components and molecular functions are shown with green, orange and blue diagrams, respectively. The KEGG enrichment of TNBS vs. control group (**D**) and MBD vs. TNBS (**E**). M, TNBS group; C, control; Br, MBD group.

Transcriptome sequencing was carried out to further study the potential mechanism of MBD against TNBS-induced IBD. The results showed that 366 genes were up-regulated and 1240 genes were down-regulated in the TNBS group compared to the control group. Furthermore, there were 149 genes up-regulated and 7 down-regulated in MBD group compared to the TNBS group. In addition, there were 514 genes up-regulated and 188 genes down-regulated in the MBD group compared to control group (Figure 7A).

Then, GO analysis was conducted on the deferentially expressed genes (DEGs) (Figure 7B,C). The results showed that changed biological processes mainly included cellular iron ion homeostasis, intracellular sequestering and the transport of iron ions in the TNBS group compared to the control group. Conversely, the changed biological processes mainly included the cellular response to xenobiotic stimuli in the MBD group compared with the TNBS group. These results indicated that MBD might inhibit TNBS-induced inflammation by regulating the pathways related to the cellular response to xenobiotic stimuli and iron metabolism and homeostasis.

The KEGG analysis results showed that NOD-like receptor signaling pathways were closely related to IBD and ferroptosis. As to MBD, the DEGs were mainly related to the NOD-like and Toll-like receptor signaling pathways, and C-type lectin receptor pathways (Figure 7D,E). These results indicated that MBD could inhibit TNBS-induced inflammation by adjusting various pathways, including the NOD-like and Toll-like receptor signaling pathways.

### 2.8. Gene Expression

**Figure 8:**
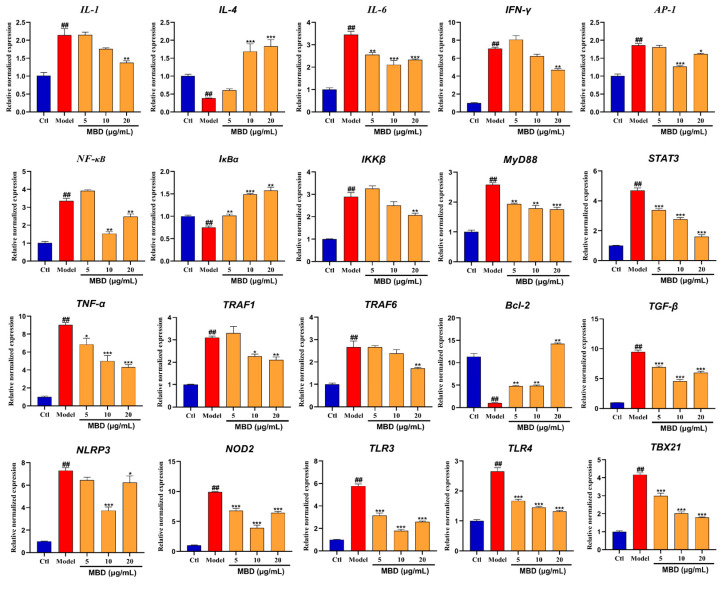
mRNA expression of genes involved in inflammation after MBD exposure. Gene mRNA expression is shown as the relative expression by fold compared to the Ctl. ## *p* ≤ 0.01 vs. Ctl; * *p* ≤ 0.05, ** *p* ≤ 0.01, *** *p* ≤ 0.01 vs. model.

To further validate the results of mRNA sequencing and network pharmacology, RT-PCR was used to test the inflammation-related genes and pathways. As shown in Figure 8, the mRNA expression of *IL1*, *IL6*, *AP1*, *IFNγ*, *NF-κB*, *IKKβ*, *MyD88*, *STAT3*, *TNF-α*, *TRAF1*, *TRAF6*, *TGFβ*, *NLRP3*, *NOD2*, *TLR3*, *TLR4* and *TBX21* was significantly enhanced by TNBS, while MBD could inhibit the elevation of these genes. However, the expression of *IL4*, *IκBα* and *Bcl-2* was significantly inhibited by TNBS, while MBD could activate the expression of these genes.

## 3. Discussion

Various drugs have been developed and used to treat IBD, including aminosalicylates, antibiotics, corticosteroids, immunosuppressive drugs and supportive medications [24]. However, there are lots of limitations, including the easy recurrence of IBD after drug withdrawal, and adverse effects, such as drug resistance, nausea, headache and diarrhea, during the treatment [25]. To date, there are still no drugs that can completely cure IBD. Therefore, it is urgent to develop green and efficient drugs to treat IBD. Here, methyl 3-bromo-4,5-dihydroxybenzoate (MBD), a bromophenol compound, which was derived from marine algae and sponges, was found to have anti-inflammatory activity. Furthermore, the anti-inflammatory activity, especially in IBD, and the underlying mechanism were investigated in a zebrafish IBD model induced by TNBS.

Various factors, including infection, radiation and mechanical injury, can lead to inflammatory responses in the body. When an inflammatory response occurs, it will cause a series of responses, including migration of the immune cell to the damage site, ROS elevation, angiogenesis and the production of inflammatory factors [26]. Here, the *Tg(zlyz:EGFP)* zebrafish line, with immune cells labeled with EGFP, was used to directly observe the migration of immune cells to inflammation sites. Previous reports have shown that CuSO_4_, LPS and tail-cutting have been widely used to induce inflammation in zebrafish by leading to the migration or accumulation of immune cells [27]. CuSO_4_ can damage the facial nerve cells of zebrafish, LPS can cause systemic inflammation, and tail-cutting can cause mechanical damage to the tail [28]. Our results showed that MBD could inhibit the inflammatory response by inhibiting the intestinal migration of immune cells induced by TNBS. These results indicated that MBD had anti-inflammatory activity in various inflammatory models including IBD.

The failure of the gut barrier is the main characteristic of IBD. When IBD occurs, intestinal goblet cells and tight junction proteins will be reduced, leading to intestinal leakage. Then, lots of harmful bacteria or viruses can enter into the intestine, multiply and further induce inflammation of the intestinal mucosa [29]. Intestinal structural integrity is closely related to intestinal function. The HE staining and TEM results indicated that intestinal structural integrity could be protected by MBD, as it protects against damage inflicted by TNBS. In addition, peristalsis and excretion capability are the key signs of intestinal function [29]. The calcein staining results showed that the peristalsis and excretion capabilities were enhanced by MBD in the zebrafish IBD model. These results indicated that MBD could protect the gut barrier and the function of the intestine in IBD.

ROS is an important signal and closely involved in the occurrence and progression of IBD. Excessive ROS can affect lipid metabolism, as well as DNA and protein synthesis, then induce the apoptosis of intestinal epithelial cells, and further damage the structure and the function of the intestine [30]. Here, the ROS staining results showed that TNBS could promote the production of ROS, which was consistent with previous reports [31,32]. On the contrary, MBD could inhibit the elevation of ROS induced by TNBS. Our results indicated that the promotion of antioxidant capacity by MBD was helpful to alleviate IBD symptoms.

Network pharmacology is a useful tool to analyze potential targets and pathways based on relevant databases. Our results indicated that MBD was closely correlated to genes such as TNF, STAT3 and EGFR, which are key markers in the inflammatory response [33,34]. In addition to the molecular docking results showing that MBD could interact with these targets, RNA sequencing was also conducted to further investigate the potential targets of MBD. The results indicated that inflammation related to various pathways, such as the NF-κB/STAT3 and TLR pathways, was changed after MBD treatment. To validate the results of the network pharmacology analysis and RNA sequencing, RT-PCR was conducted after MBD treatment in zebrafish. The results showed that *TNF*, *IL1*, *IL6*, *IFNγ* and *MyD88*, key indicators of inflammation [35], were remarkably decreased after MBD treatment compared to the TNBS group. The *NF-κB/STAT3* pathway could be down-regulated by MBD, consistent with the results of network pharmacology and RNA Seq. Toll-like receptor (TLR) pathways are closely correlated to IBD [36,37]. When the gut barrier is damaged, the products from pathogens or microorganisms can bind TLRs to induce inflammation by activating the innate immune system and then activating the *NF-κB* pathway [38,39]. In our study, TLRs pathways, including *TLRs* (*TLR3*, *TLR4*), *MyD88*, *TRAF1* and *TRAF6*, were inhibited by MBD. These results indicated that MBD could alleviate IBD by inhibiting the NF-κB/STAT3 and TLR pathways.

To summarize, methyl 3-bromo-4,5-dihydroxybenzoate (MBD), a bromophenol compound derived from marine organisms, could alleviate inflammatory bowel disease (IBD). MBD could protect the structural intensity of the gut barrier and the function of the intestine against the damage caused by TNBS in zebrafish. The potential molecular mechanisms involve the regulation of the NF-κB and TLR pathways. Overall, our results suggest that MBD has the potential to alleviate IBD by inhibiting inflammation-related pathways.

## 4. Materials and Methods

### 4.1. Zebrafish Strains and Culture

*TG(zlyz:EGFP)* and wild-type (WT) AB were maintained on the Zebrafish Drug Screening Platform of Shandong Academy of Science and cultured at 28 ± 0.5 °C in an automatic zebrafish housing system with a 14/10 h light/dark photoperiod. The zebrafish were fed twice daily with newly hatched brine shrimps. Fish spawning, embryo egg collection and larva culture were carried out according to standard procedure [16]. Healthy embryos/larvae from 48 to 96 hpf were used for the subsequent experiments.

### 4.2. Chemical Reagent

MBD (CAS No.: 65841-10-3, >99.5%) was obtained from TargetMol Biochemical Technology Co., (Shanghai, China). It was prepared in DMSO at a concentration of 20 mM and stored in a −20 °C refrigerator. Then, the working solution was prepared in ddH_2_O. All the reagents and chemicals were of analytical grade.

### 4.3. Inflammation Induced by CuSO_4_, Tail Amputation, LPS or TNBS

Acute inflammation was induced by CuSO_4_ as previously described [19]. Briefly, *Tg(zlyz:EGFP)* zebrafish larvae at 72 hpf (10 tails each group) were pretreated with MBD (5, 10, 20 μM) or indomethacin (20 μM) for 2 h. Then, CuSO_4_ was added and treated for 1 h at a concentration of 20 μM. Then, microscopy was conducted, focusing on an indicated area with migrated immune cells, and the number of cells was used for analysis. Indomethacin served as the positive control.

After zebrafish were anesthetized in 0.04% tricaine, tail amputation was carried out on Petri dishes with 2% agarose under a stereomicroscope. Then, the zebrafish (10 tails in each group) were incubated with indomethacin (20 μM) and MBD (5, 10, 20 μM). Fish water with 5‰ DMSO served as the control and the fish who did not undergo tail-cutting served as the negative control. After 6 h, microscopy was conducted, focusing on the cutting area, and the immune cells that had migrated to the damaged area were analyzed using ImageJ 1.5.1 software [40,41].

A systemic inflammation model was established in zebrafish through LPS treatment. Briefly, *Tg(zlyz:EGFP)* was incubated with LPS with/without 5-aminosalicylic acid (5-ASA, 20 μM) and/or MBD (5, 10, 20 μM) for 24 h from 72 hpf to 96 hpf. A photograph was taken under a fluorescence microscope. The fluorescence intensity was analyzed using ImageJ software.

The IBD zebrafish model was established using TNBS as previously reported [24]. Briefly, the zebrafish larvae (10 tails per group) were incubated with 50 μg/mL TNBS from 72 hpf to 124 hpf to establish the IBD model. Then, 5-ASA (20 μM) or MBD (5, 10, 20 μM) was added and incubated for another 24 h. Then, the intestinal peristalsis frequency per minute was counted under a microscope and a microscopy image was taken. The immune cells that had migrated to the intestinal region were used for analysis with ImageJ software.

### 4.4. Intestinal Efflux Efficiency Detection

The intestinal efflux efficiency was detected based on the IBD model induced by TNBS using a previously reported method [27]. Briefly, after the IBD model was established, the zebrafish were treated with calcein solution for 1.5 h and then rinsed with water 3 times. Microscopy was carried out and then the zebrafish larvae were cultured for an additional 16 h in the dark. Then, a microscopy image was taken again for the second time, and the integrated optical density (IOD) of the calcein dye staining was analyzed using ImageJ software. The calculation formulae were as follows.

IOD_Ctl_ = [IOD_CTL0_ − IOD_CTL1_]/IOD_CTL0_

IOD_TNBS_ = [IOD_TNBS0_ − IOD_TNBS1_]/IOD_TNBS0_

### 4.5. Histopathological Analysis

To check the pathological changes in the intestine, HE staining and transmission TEM were conducted following a previously described method [27]. Briefly, after treatment, the zebrafish were fixed in 4% PFA, dehydrated with ethanol of gradient concentration, and finally embedded in paraffin. The fixed zebrafish were sectioned and then HE staining was performed. Images of the sections with HE staining were obtained. The sections were treated with uranyl acetate and lead citrate and then electron microscopy was conducted to obtain the pictures (Hitachi HT7800, Tokyo, Japan).

### 4.6. ROS Analysis

The ROS level in the zebrafish larvae was assessed using an ROS probe, 2′-7′-Dichlorodihydrofluorescein diacetate (DCFH-DA), which reacted with ROS and changed to a fluorescent dichlorofluorescein (DCF). After treatment, the zebrafish larvae (10 tails per group) were stained with 30 µg/mL DCFH-DA solution in the dark for 1 h. After the zebrafish were anesthetized, a microscopy image was taken using a fluorescence microscope. The intensity of fluorescence was used to calculate the ROS level.

### 4.7. Network Pharmacology Analysis Methods

Network pharmacology analysis was conducted according to the previous method [30,31]. Briefly, for the target fish, the sdf format of MDB was unloaded to the Swiss Target Prediction database and PharmMapper database. Homo sapiens was set up as the screening species. The collected target proteins were used to establish a protein–protein interaction (PPI) network in the String platform: “https://string-db.org/ (accessed on 2 April 2024)”.

For the GO and KEGG analyses, the targets, which were shared by MBD and IBD, were used for annotation, visualization and integrated discovery in the DAVID database: “https://david.ncifcrf.gov/ (accessed on 2 April 2024)”. *p* < 0.05 was set up with the screening conditions of the GO and KEGG analyses.

The data of the correlation of MBD and IBD targets and the pathways were filed into excel and then the target and pathway (T-P) networks were established in Cytoscape 3.6.1 software. The PPT network was also created using Cytoscape 3.6.1.

For the molecular docking, the first 8 potential targets (ALB, TNF, EGFR, SAC, ESR1 STAT3, BCL2, CASP3) in PPI were chosen for further analysis as previously reported [33,34]. Firstly, the 3D structures of the targets were established and energy minimized in Chem3D Pro14.0 software. Pymol 2.6.0 software was used to obtain an interaction picture of MBD and the target proteins.

### 4.8. Transcriptomic Analysis

mRNA extraction, cDNA synthesis, RNAseq and transcriptomic analysis were conducted using previously reported methods [42]. After MBD treatment, zebrafish larvae (20 tails per group) were gathered and subjected to RNAseq by the OE Biotechnique Company (Shanghai, China). The differentially expressed genes (DEGs) were defined with a fold change >1.5 and *p*-value (padj) < 0.05. And the top 20 DEGs were used for further analysis. GO and KEGG analysis were performed using OECloud tools.

### 4.9. Real-Time Quantitative PCR (qRT-PCR)

qRT-PCR was performed to detect the mRNA expression of inflammation-related genes using the SYBR Green mix (Takara, Dalian, China). The 2^−ΔΔCt^ method was employed to analyze the comparative expression of the detected genes. The housekeeping gene β-actin was used as the internal control. The primers used in the experiment were provided by Boshang Bioengineering Company (Shanghai, China) and are listed in Table 1.

### 4.10. Statistical Analysis

The data were analyzed with GraphPad Prism 5.0 software, using the one-way ANOVA method, combined with Dunnett’s post hoc t-test. At least three independent experiments were conducted, and all of the data are shown as the mean ± SD. A *p* value < 0.05 was recognized as statistically significant (# and * *p* ≤ 0.05, ## and ** *p* ≤ 0.01, *** *p* ≤ 0.001).

## Figures and Tables

**Table 1:** Primer sequences.

Gene	Forward (5′-3′)	Reverse (5′-3′)
*β-actin*	AGAGCTATGAGCTGCCTGACG	CCGCAAGATTCCATACCCA
*IL-1*	AGGTGCATCGTGCACATAAG	AAGCTGATGGCCCTAAACAG
*IL-4*	GCCATATCCACGGATGCGACAA	GGTGTTCTTCGTTGCTGTGAGGA
*IL-6*	TCTGCTACACTGGCTACA	ACATCCTGAACTTCGTCTC
*NF-κB*	CAATGAAATCTCCTGGGTG	CAATGAAATCTCCTGGGTG
*TNF-α*	ATGAGCACAGAAAGCATGATC	TACAGGCTTGTCACTCGAATT
*STAT3*	CAGCAGCTTGACACACGGTA	AAACACCAAAGTGGCATGTGA
*AP-1*	CCACCGCTCTCTCCTATC	ATCCTCTCCAGTTTCCTCTT
*NLRP3*	AGCCTTCCAGGATCCTCTTC	CTTGGGCAGCAGTTTCTTTC
*TLR3*	TTGCCTTGTATCTACTTTTGGGG	TCAACACTGTTATGTTTGTGGGT
*TLR4*	AGACCTGTCCCTGAACCCTAT	CGATGGACTTCTAAACCAGCCA
*MyD88*	ATCCACAGGGACTGACACC	CCACCACCATCCTCTTACAC
*IκBβ*	GGTGGAAAGACTCCTGAAAGC	TGTAGTTAGGGAAGGTAAGAATG
*IKKβ*	ACTCTCAGCTCAGTAAGACCG	CCACAGTCTTCTCATCCTCGTT
*TRAF1*	GGGCAACCCAGACAAAGT	CATCGTGGAGGCTGAAGG
*TRAF6*	GCCCATGCCGTAT	ACTGAATGTGCAGGGGACTG
*NOD2*	TGCCTCGGGAACAGTAAGAC	GCCGCCCTCTCCATTAAAC
*IFN-γ*	CTCGTAAGACTCCTTGTGT	ATGAACTCGGTGAACTGG
*Bcl-2*	TCACTCGTTCAGACCCTCAT	ACGCTTTCCACGCACAT
*TGF-β*	GAACTCGCTTTGTCTCCA	TACAGTCGCAGTATAACCTCA
*TBX21*	CTCACCAACCATACCTCTC	TGTATTCGGTCTCGTAAGC

## Data Availability

The data shown in the current research are available upon request from the corresponding author.
